# Stabilized Amorphous Calcium Carbonate as a Precursor of Microcoating on Calcite

**DOI:** 10.3390/ma13173762

**Published:** 2020-08-26

**Authors:** Taeyoung Jeon, Ye-Eun Na, Dongchan Jang, Il Won Kim

**Affiliations:** 1 Department of Chemical Engineering, Soongsil University, Seoul 06978, Korea; mogistto@gmail.com; 2 Department of Nuclear and Quantum Engineering, KAIST, Daejeon 34141, Korea; skdpdms94@kaist.ac.kr

**Keywords:** calcium carbonate, amorphous precursor, calcite, epitaxy, macromolecule

## Abstract

Highly controlled biomineralization of calcium carbonate is via non-classical mesocrystallization of amorphous precursors. In the present study, a simple in vitro assay was developed to mimic the biological process, which involved stabilized amorphous calcium carbonate and a single crystal substrate of calcite. The microcoating layer formed on the calcite substrate displayed mesocrystalline characteristics, and the layers near the substrate were strongly influenced by the epitaxy to the substrate. This behavior was preserved even when the morphology of the coating layer was modified with poly(acrylic acid), a model anionic macromolecule. Interestingly, the extent of the epitaxy increased substantially with poly(ethylene imine), which barely affected the crystal morphology. The in vitro assay in the present study will be useful in the investigations of the biomineralization and bioinspired crystallization of calcium carbonate in general.

## 1. Introduction

Natural composite materials, such as woods, bones, teeth, and mollusk shells, exhibit superior mechanical properties, originating from their intricately hierarchical structures [1,2]. Among these, mollusk shells and tooth enamel possess high amounts of inorganic crystals (>95%) without showing brittle failure behavior [3,4]. The high toughness coupled with their intrinsic hardness is the ideal combination for their everyday functions.

Mollusk shells have been extensively studied as a model system of high-performance composites. Their hierarchical structures of nanograin and microplate have been well documented [3,5,6,7]. The structural construction of the major inorganic phase (calcium carbonate) was via minor organic components, and amorphous calcium carbonate (ACC) is an essential precursor during the control process, which transforms into desired polymorphs and morphologies of crystals most likely through mesocrystallization [7,8,9,10,11,12].

In vitro crystallization of calcium carbonate has been instrumental in elucidating the probable role of the constituents of biominerals and developing useful approaches to generate biomimetic and bioinspired composites. The crystallization usually starts by simply increasing the supersaturation of aqueous solutions, and additives and substrates are tested in the system to identify their effects. While there are too diverse examples to cover in this report, some early examples include the investigations employing biomacromolecules extracted from biominerals to understand their roles as additives and substrates [13,14,15]. Additionally, simple synthetic materials have been studied to understand the physicochemical principles of the morphological and polymorphic controls of calcium carbonate [16,17].

As the importance of ACC is becoming evident, diverse methods have been devised to stabilize ACC and examine its unique role in the nonclassical crystallization. The stabilization is usually done with the addition of organic agents, such as ethanol, citrate, and other anionic molecules, which makes it cumbersome to investigate the effects of additional additives or substrates using the stabilized systems [18,19,20,21,22,23]. On the other hand, the lifetime of ACC in aqueous environments without the stabilizing agents is too short (often less than 1 min) to be useful unless at low temperature (<10 °C) or highly basic (pH > 11) conditions [20,24,25].

The purpose of our study is to develop a simple in vitro assay of mesocrystallization that can be used with diverse additives. The high pH method was modified to further stabilize ACC without the addition of stabilizing agents, and the surface of calcite single crystal was employed to initiate the assembly of ACC and the subsequent mesocrystallization [26]. As a result, a few micron thick coating layers were constructed, and their microstructures were analyzed in the presence and absence of model macromolecular additives.

## 2. Materials and Methods

### 2.1. Materials

Sodium carbonate (Na_2_CO_3_, >99.0%), calcium chloride (CaCl_2_, >97%), and a 0.50 M NaOH aqueous solution were purchased from Sigma-Aldrich (St. Louis, MO, USA). Poly(acrylic acid) (PAA, M_w_ 1800) and poly(ethylene imine) (PEI, M_w_ 1300) were also from Sigma-Aldrich. Acetone (>95%) was obtained from Samchun Chemical (Seoul, Korea). Deionized water (DI water, resistivity >18.2 MΩ·cm) was from a Direct-Q3 water purification system of Millipore (Burlington, MA, USA). Calcite (geological CaCO_3_) was acquired from Hansol Education Co. (Seoul, Korea).

### 2.2. Preparation and Characterization of Stabilized ACC

Stabilized ACC was obtained by mixing an equal volume (50 mL) of 0.10 M CaCl_2_ (aq) and 1.0 M Na_2_CO_3_ (aq) at the initial pH 13. The initial pH of the mixture was adjusted by adding a proper amount (30 mL) of 0.50 M NaOH (aq). First, individual solutions were prepared and kept below 5 °C in a refrigerator. Second, the CaCl_2_ (aq) was mixed into the premixed solution of Na_2_CO_3_ (aq) and NaOH (aq) at room temperature. Finally, the precipitated mixture was filtered after the desired duration using a suction filtration system (No. 20 filter paper, Hyundai Micro, Seoul, Korea), and it was successively washed with DI water (7 mL) and acetone. The filtrate was dried in a vacuum oven (J-DVO1, JISICO, Seoul, Korea) at 60 °C for at least 12 h before further characterization.

The dried powders were analyzed using powder X-ray diffraction (XRD) and scanning electron microscopy (SEM). XRD was performed with a D2 PHASER from Bruker AXS (Billerica, MA, USA) using CuKα radiation (λ = 1.5406 Å) at 30 kV and 10 mA. A 2θ range of 15 to 55° was scanned at a rate of 1°/min (0.02° increment). A zero-background holder (Bruker AXS) was used to minimize background noise. SEM observation was conducted using a JEOL JSM-6360A microscope (Tokyo, Japan) after thin Au coating (Cressington Sputter Coater 108, Watford, UK) to minimize charging.

The phase change of the solid within the mixture was also studied in situ via optical microscopy (OM: BX51, Olympus, Tokyo, Japan) in the transmission mode. One mL of the just mixed solution was transferred on a glass slide and immediately blanketed with a cover glass. The change in birefringence was monitored under cross-polarization equipped with a first-order retardation plate.

### 2.3. Formation of the Coating Layers on Calcite Single Crystals

A typical experiment with stabilized ACC to form a coating layer of calcium carbonate on the surface of a calcite single crystal is described as follows. First, a single crystal of calcite (a few mm on each side) was cleaved from a large single crystal block of geological calcite. Second, it was fixed on a circular cover glass with a UV curable adhesive. After curing, the sample was immersed into the mixed solution of Na_2_CO_3_ (aq) and NaOH (aq), and CaCl_2_ (aq) was added to start the coating formation. The solution composition was as described in the previous section.

When a polymeric additive (PAA or PEI) was used, it was pre-dissolved in the CaCl_2_ (aq) solution. After the desired duration, the calcite sample was taken out of the solution, and it was washed with DI water (7 mL), blow-dried with nitrogen gas, and dried at 60 °C in a convection oven (OF-12G, Jeio Tech, Daejeon, Korea).

The coating layers were studied with SEM (see the previous section for the equipment and procedure). The coating surfaces (top view) were observed as they were, and the cross-sections (side view) after fracture by a razor blade.

### 2.4. Microstructural Analysis of the Coating Layers

The cross-sectional analyses on the microstructure and crystallographic orientation of the coating layers were conducted using a transmission electron microscope (TEM: JEM 2100F HR, JEOL, Tokyo, Japan) under the diffraction contrast mode. The cross-sectional TEM specimens were prepared by lifting out the rectangular block from the bulk materials, transferring to the TEM grids, and sequentially thinning with the focused ion beam (FIB) milling until they become electron-transparent. To investigate the crystallographic orientation relationship between the substrate and the coated layers, we first aligned the specimen along a zone axis of the single-crystalline substrate and then collected the electron diffraction patterns of the deposited layers after moving the areas of interest under the electron beam without changing the stage tilting angles and beam conditions.

## 3. Results and Discussion

### 3.1. ACC Formation and Characterization

**Figure 1:**
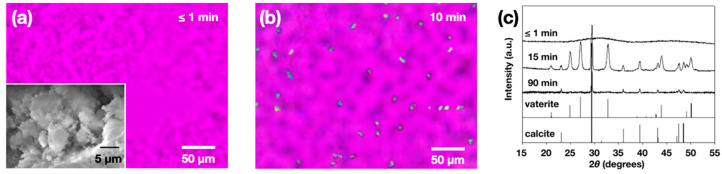
Optical micrographs under cross polarization right after ACC formation (**a**) and after 10 min (**b**); a SEM image of ACC before transformation was shown as an inset of (**a**). XRD patterns (**c**) of ACC and its transformed phases with time were shown along with the reference peaks of vaterite and calcite [28].

ACC formation under the conditions employed in the present study was confirmed using OM and XRD. The solid phase obtained right after the mixing of CaCl_2_ (aq) and Na_2_CO_3_ (aq) did not show birefringence when observed under cross-polarization (Figure 1a). In contrast, the birefringence after 10 min indicated the formation of crystalline phases (Figure 1b). The initial appearance of the birefringence was after about 5 min. Note that the ACC particles appear ca. 100 to 200 nm in their size (Figure 1a inset, SEM micrograph).

The XRD results are in agreement with OM observations (Figure 1c). An amorphous halo was the only characteristic of the initial solid phase (≤1 min). After 15 min, diffraction peaks characteristic to vaterite and calcite phases appeared. After 90 min, calcite peaks were the sole contribution, and the vaterite peaks disappeared. Similar behavior has been seen for the solution crystallization of calcium carbonate, where metastable vaterite was the initial crystalline phase eventually transforming into stable calcite through a dissolution-and-recrystallization process [27,28].

In the classic high pH method, exclusive ACC preparation was possible if the entire preparation steps were at 4–5 °C [23,25]. Since the low-temperature requirement restricts the wide adaptation of the method, we modified the procedure by simply increasing the ratio of initial [CO_3_^2−^]/[Ca^2+^]. We tested the ratio from 1 to 10 for the mixing at room temperature and chose 10 for the stability of ACC (data not shown). This is in accordance with the previous studies, where high carbonate concentration slowed the growth or the precipitation of calcite [29,30]. Stoichiometrically unbalanced compositions could make the incorporation of the minor species (calcium ion in the present study) into crystal growth sites difficult by reducing its attachment frequency due to its low concentration exacerbated by the unfavorable concentration gradient near the growing surface [29,30]. We also note here that when pH was 10, ACC could not be stabilized and, therefore, isolated in pure form.

Overall, ACC stable for about 5 min at room temperature was formed by simply mixing CaCl_2_ (aq) and Na_2_CO_3_ (aq) at pH 13 and the initial [CO_3_^2−^]/[Ca^2+^] = 10. A simple but clear advantage of the current experimental condition over the precedent methods is the extended lifetime of ACC at room temperature without any organic additive. This makes the current method ideal in studying the effects of additives on the biomineralization and bioinspired crystallization mediated by ACC.

### 3.2. ACC on Calcite Substrates

**Figure 2:**
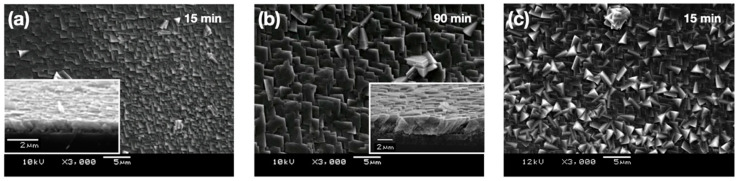
SEM images of the coating layers formed via ACC on single crystals of calcite (top down view) after 15 min (**a**) and 90 min (**b**); cross-sections of the coating layers after fracture were shown as inset images. A SEM image of the coating layer formed without ACC stabilization (**c**): top down view after 15 min.

The stabilized ACC was tested as a precursor of bioinspired crystallization of calcium carbonate on the substrate of calcite single crystal. Figure 2 shows the overgrown layers on top of freshly cleaved {104} surfaces of calcite single crystals. The morphologies of the overgrown crystals show rhombohedral facets typical with calcite. Those formed with stabilized ACC appeared to have some orientational regularities with each other, and their facets looked more or less parallel to the surfaces of the substrate single crystals (Figure 2a,b). In contrast, when formed at pH 10 without ACC stabilization, the resulting overgrown crystals displayed unsystematic orientations with each other and with the substrate (Figure 2c).

**Figure 3:**
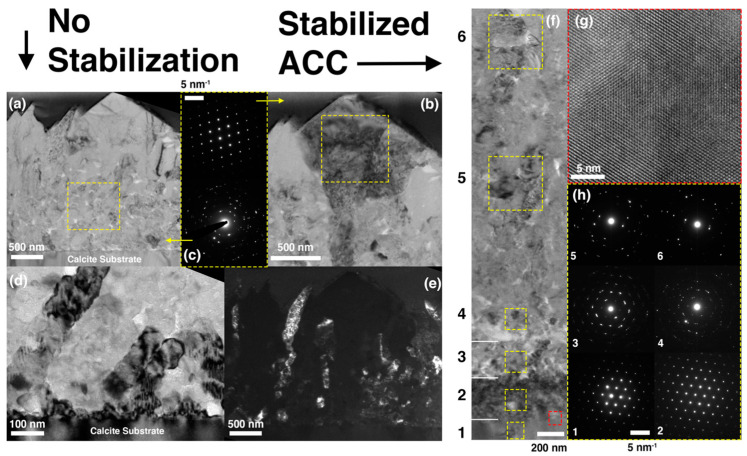
The microstructural and crystallographic characteristics of the coatings grown without (**a**–**e**) and with (**f**–**h**) the stabilized ACC, respectively. (**a**–**b**) The bright-field TEM images showing the entire coating cross section of the sample without ACC and corresponding selected area electron diffraction patterns obtained within the dotted boxes. The image in (**b**) was taken after aligning the sample along the zone axis of the grain having the faceted surface. The bright-field (**d**) and dark-field (**e**) TEM images showing the microstructures near the coating-substrate interface of the sample without ACC. (**f**) The bright-field TEM image showing the entire cross section of the sample with the stabilized ACC and (**h**) corresponding selected area electron diffraction patters obtained in the area within each dotted yellow box in (**f**). (**g**) The high-resolution TEM image taken on the coating-substrate interface in the region marked by the dotted red box in (**f**). In order to investigate the epitaxial relationship of the coated layer, the electron diffraction patterns in (**h**) were obtained without adjusting the specimen tilting angles once they are fixed along the zone axis of the substrate.

The TEM micrographs in Figure 3 clearly show the differences in the microstructural and crystallographic features between calcium carbonate coating layers grown without a–e and with f–h ACC stabilization. The cross-sectional TEM bright-field images in Figure 3a,b reveal that the faceted features on the top surface of the sample without ACC stabilization in Figure 2c are not the consequence of the epitaxy to the single-crystalline calcite substrate. Instead, the columnar grains with a width of about 100 nm (Figure 3d,e) form in the immediate vicinity of the substrate. The electron diffraction pattern in Figure 3c indicates the stochastic nature of their crystallographic orientation. During the further growth, some of the columnar grains begin growing abnormally, resulting in the formation of facets on the layer top surface (Figure 3b). The electron diffraction pattern in Figure 3c confirms that those facets are single-crystalline.

In sharp contrast, the coating layer synthesized with the stabilized ACC consists of many sub-layers whose crystallographic features gradually change from the perfect epitaxy in sub-layer 2 to randomly-oriented nano-crystallites in sub-layers 5 and 6 (see the bright-field TEM image in Figure 3f and corresponding electron diffraction patterns in Figure 3h). The high-resolution transmission electron microscope (HREM) image in Figure 3g that magnifies the interfacial region marked by the dotted red box in Figure 3f additionally supports the epitaxial growth of sub-layer 2. The electron diffraction patters for sub-layers 3 and 4 show the intermediate crystallographic characteristic between the single-crystalline epitaxy and random nanocrystals, in which the agglomerated diffraction spots suggest the existence of textured grains. The decreased crystallographic correlation away from the single crystal substrate was as expected since the crystallization of ACC was initiated at once the aqueous mixture was prepared whereas the effect of the substrate was sequential from the sublayer adjacent to the substrate [31]. We also emphasize that the lack of columnar grains suggests the importance of the ACC stabilization in non-classical mesocrystallization.

Overall, the combined observations made with SEM and TEM clearly show that the ACC stabilization enables the controlled crystallization of calcium carbonate. Two important characteristics of the controlled crystallization are the epitaxy to the substrate and the mesocrystallization of ACC, both of with are key phenomena observed in biomineralization [3,7,8,9].

### 3.3. Effects of Model Macromolecules

**Figure 4:**
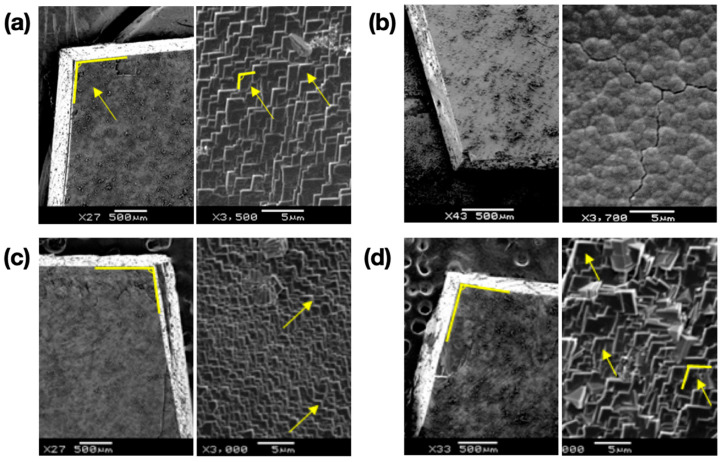
SEM images of the coating layers formed via ACC on single crystals of calcite (top down view) after 90 min with 0.04 mM PAA (**a**); 0.4 mM PAA (**b**); 0.04 mM PEI (**c**); 0.4 mM PEI (**d**).

The ACC/calcite growth system was tested with two model macromolecular compounds: PAA and PEI. PAA is known as a strong morphology modifier, whereas PEI is generally considered ineffective [32,33,34]. Figure 4 shows the overgrown layers on top of freshly cleaved calcite surfaces in the presence of the macromolecular additives. Zoom-out images were shown on the left side for all cases to show the facet orientation of the single crystal substrates; zoom-in images on the right side were obtained without changing the stage orientations. When the additive concentration was 0.04 mM, the typical calcite morphology was nearly preserved (Figure 4a for PAA and 4c for PEI). When the concentration of PAA was increased to 0.4 mM, the morphology modification of calcite was obvious (Figure 4b). Typical rhombohedral facets of calcite were replaced with round features, which was in agreement with the previous studies [32]. We note here that further ACC stabilization, which had been observed in other studies, was not evident in the current experiment, most likely because of the presence of the calcite substrate acting as the initial point of crystallization [35,36]. In contrast, the rhombohedral facets were largely preserved with 0.4 mM PEI (Figure 4d), although the feature size increased compared to the case of 0.04 mM PEI. The increased feature size with the higher PEI concentration was most likely due to the increase of solution viscosity, which could affect the crystallization kinetics in such a way that the growth of larger crystals was promoted, an extreme case being crystal growth in gels [37,38].

**Figure 5:**
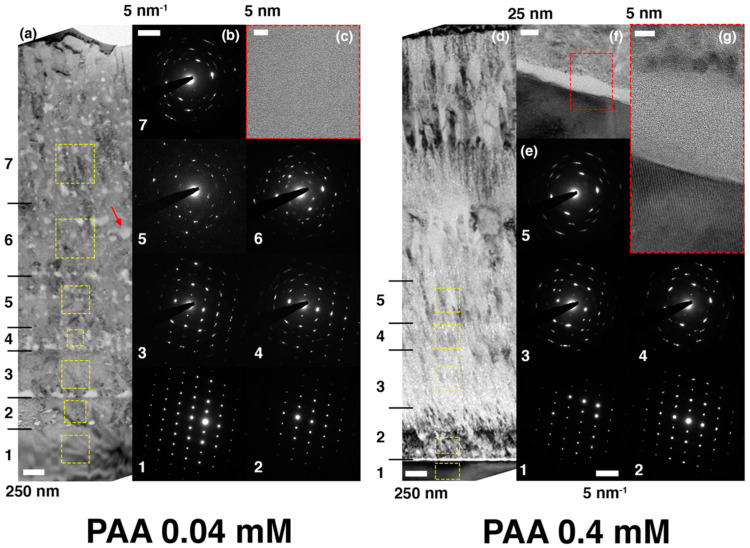
The microstructural and crystallographic characteristics of the coatings grown with (**a**–**c**) 0.04 mM and (**d**–**g**) 0.4 mM of PAA, respectively. (**a**) The bright-field TEM image showing the entire coating cross section of the sample with 0.04 mM of PAA and (**b**) corresponding selected area electron diffraction patterns obtained in the area within each dotted yellow box in (**a**). (**c**) The high-resolution TEM image taken on the brightly-contrasted spot indicated by the red arrow in (**a**). (**d**) The bright-field TEM image showing the entire coating cross section of the sample with 0.4 mM of PAA and (**e**) corresponding selected area electron diffraction patterns obtained in the area within each dotted yellow box in (**d**). (**f**) The bright-field TEM image showing the interfacial region between the substrate and coating. (**g**) The high-resolution TEM image magnifying the region within the red dotted box in (**f**). In order to investigate the epitaxial relationship of the coated layer, the electron diffraction patterns in (**b**) and (**e**) were obtained without adjusting the specimen tilting angles once they are fixed along the zone axis of the substrate.

Figure 5 shows the cross-sectional TEM micrographs for the coated layers grown with 0.04 mM (a through c) and 0.4 mM (d through g) of PAA, together with the electron diffraction patterns collected from various regions. Noticeably, both coatings consist of many sub-layers across which the crystallographic orientation changes to some extent. As evidenced in the electron diffraction patterns in Figure 5b, the epitaxy more or less persists from the substrate-coating interface to sub-layer 4 in the sample synthesized with 0.04 mM of PAA, even though a slight misorientation appears in sub-layers 3 and 4, indicated by the elongation of each diffraction spot. The single-crystal-like features in the diffraction patterns disappear entirely on the sub-layers above 5. The TEM image in Figure 5a shows that the features of mesocrystallization (and lack of the columnar grains) are preserved even with the addition of PAA. Interestingly, it also exhibits many regions with bright contrast, most of which line up horizontally. The HREM image in Figure 5c taken on one of such features (indicated by the red arrow in Figure 5a) reveals that they are the amorphous phase embedded within the crystalline matrix. We presume that the macromolecular additive, pushed away from ACC during ACC-to-crystal transformation, is accumulated in this region to locally thwart the further transformation of ACC. This hypothesis is yet to be scrutinized.

On the contrary, the coated layer with 0.4 mM of PAA shows the distinguished microstructures. While the evidence of mesocrystallization is obvious, the nanograins in the sublayers look smaller and often fibrillar. Besides, unlike the epitaxial interface between the substrate and the first sub-layer in the 0.04 mM PAA sample, there exists a thin amorphous layer with a thickness of ca. 20 nm right on top of the substrate in the 0.4 mM PAA one (Figure 5f,g). Nonetheless, as is obvious when comparing the diffraction patterns 1 and 2 in Figure 5e, the first sub-layer of this sample has an almost identical crystallographic orientation with the substrate, which implies the porous or patchy nature of the amorphous layer when the entire substrate surface was considered [39,40]. However, the single-crystallinity vanishes in the subsequent sub-layers; the extent of epitaxy is comparable to that without additive (Figure 3).

**Figure 6:**
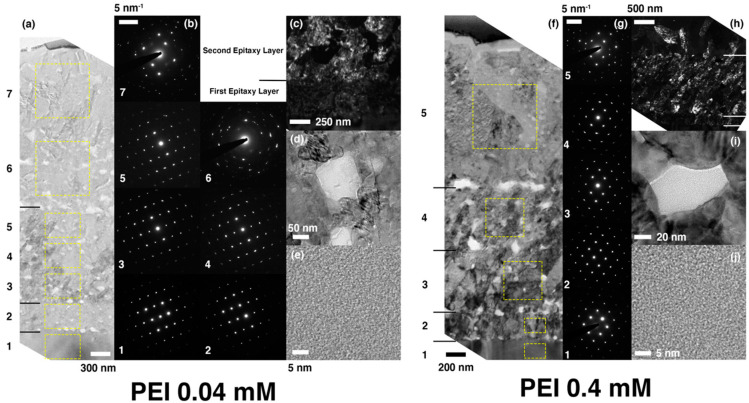
The microstructural and crystallographic characteristics of the coatings grown with (**a**–**e**) 0.04 mM and (**f**–**j**) 0.4 mM of PEI, respectively. (**a**) The bright-field TEM image showing the entire coating cross section of the sample with 0.04 mM of PEI and (**b**) corresponding selected area electron diffraction patterns obtained in the area within each dotted yellow box in (**a**). (**c**) The dark-field TEM image showing the first few sub-layers above the substrate. (**d**) The bright-field TEM image showing one of the brightly-contrasted spots in (**a**). (**e**) The high-resolution TEM image magnifying the bright spot in (**d**). (**f**) The bright-field TEM image showing the entire coating cross section of the sample with 0.4 mM of PEI and (**g**) corresponding selected area electron diffraction patterns obtained in the area within each dotted yellow box in (**f**). (**h**) The dark-field TEM image showing the first a few sub-layers above the substrate. (**i**) The bright-field TEM image showing one of the brightly-contrasted spots in (**f**). (**j**) The high-resolution TEM image magnifying the bright spot in (**i**). In order to investigate the epitaxial relationship of the coated layer, the electron diffraction patterns in (**b**) and (**g**) were obtained without adjusting the specimen tilting angles once they are fixed along the zone axis of the substrate.

Figure 6 shows the cross-sectional TEM micrographs for the coated layers grown with 0.04 mM (a through e) and 0.4 mM (f through j) of PEI, together with the electron diffraction patterns collected from various regions. Qualitatively, the overall microstructural features of both coatings look similar to those in the 0.04 mM PAA sample, showing the evidence of mesocrystallization via ACC transformation. All of them are composed of many sub-layers out of which the first several ones have the epitaxial relationship with the substrate. Additionally, as shown in Figure 6d,e,i,j, they commonly contain the amorphous inclusions within the crystalline matrices. Quantitatively, the extent of epitaxy away from the substrate increased with PEI. Figure 6 shows that the crystallographic correlations are well sustained almost up to 2 μm, and even top layers show some orientational correlations with the substrate 3–4 μm apart. This behavior is not seen in other cases in the present study. The crystallographic correlations are not well defined over ca. 0.5 μm without additive and with 0.4 mM PAA, and ca. 1.2 μm with 0.04 mM PAA (Figure 3 and Figure 5). We believe that the nature of PEI weakly interacting with calcium carbonate may play a role in stabilizing ACC, most likely through gentle encapsulation to eventually enable the extended influence of the substrate [41,42]. The exact role of PEI is under investigation in terms of the kinetics of ACC transformation and the distribution of the macromolecules.

## 4. Conclusions

In summary, a simple in vitro assay for the crystallization of calcium carbonate was established with the use of the stabilized ACC (pH 13 and [CO_3_^2−^]/[Ca^2+^] = 10) and the calcite single-crystal substrate. The current assay produced a few micrometer-thick crystalline layers on the substrate, and microstructural observations revealed their mesocrystalline nature via ACC transformation—even displaying embedded ACC. The crystallographic orientation of the coating layer exhibited the epitaxial control originating from the single crystal substrate. The extent of the epitaxy along with the distance away from the substrate increased with the addition of the macromolecular additives, more so with the less interacting PEI. In contrast, PAA influenced the morphology of the coating layer globally as well in the level of the constituting nanograins. An investigation is currently in progress to further understand the aspects of mesocrystallization, such as the kinetics of ACC transformation and the distribution of the macromolecular additives, and to correlate the mechanical properties of the coating layers with their microstructures. The current method can be widely used for the biomimetic and bioinspired crystallization of calcium carbonate, especially with various organic additives and inorganic substrates [12,17,40,43]. Additionally, the current results could have implications in diverse applications, such as drug delivery and limestone-structure restoration [44,45,46].
